# Measurement and mapping of maternal health service coverage through a novel composite index: a sub-national level analysis in India

**DOI:** 10.1186/s12884-022-05080-5

**Published:** 2022-10-10

**Authors:** Tanvi Kiran, K. P. Junaid, Vineeth Rajagopal, Madhu Gupta, Divya Sharma

**Affiliations:** Department of Community Medicine and School of Public Health, Post Graduate Institute of Medical Education and Research (PGIMER), Chandigarh, India

**Keywords:** Maternal health, Service coverage, Indian states, Composite index, Antenatal care, Intrapartum care, Postnatal care

## Abstract

**Background:**

Expansion of maternal health service coverage is crucial for the survival and wellbeing of both mother and child. To date, limited literature exists on the measurement of maternal health service coverage at the sub-national level in India. The prime objectives of the study were to comprehensively measure the maternal health service coverage by generating a composite index, map India by categorizing it into low, medium and high zones and examine its incremental changes over time.

**Methods:**

Utilising a nationally representative time series data of 15 key indicators spread across three domains of antenatal care, intranatal care and postnatal care, we constructed a novel ‘Maternal Health Service Coverage Index’ (MHSI) for 29 states and 5 union territories of India for the base (2017–18) and reference (2019–20) years. Following a rigorous procedure, MHSI scores were generated using both arithmetic mean and geometric mean approaches. We categorized India into low, medium and high maternal health service coverage zones and further generated geospatial maps to examine the extent and transition of maternal health service coverage from base to reference year.

**Results:**

India registered the highest mean percentage coverage (93.7%) for ‘institutional delivery’ and the lowest for ‘treatment for obstetric complications’ (9.3%) among all the indicators. Depending on the usage of arithmetic mean and geometric mean approaches, the maternal health service coverage index score for India exhibited marginal incremental change (between 0.015—0.019 index points) in the reference year. West zone exhibited an upward transition in the coverage of maternal health service indicators, while none of the zones recorded a downward movement. The states of Mizoram (east zone) and the Union Territory of Puducherry (south zone) showed a downward transition. Union territories of Dadra & Nagar Haveli (west zone) and Chandigarh (north zone), along with the states of Maharashtra (west zone), Assam, as well as Jharkhand (both from the east & north east zone), showed upward transition.

**Conclusion:**

Overall, maternal health service coverage is increasing across India. Our study offers a novel summary measure to comprehensively quantify the coverage of maternal health services, which can momentously help India identify lagged indicators and low performing regions, thereby warranting the targeted interventions and concentrated programmatic efforts to bolster the maternal health service coverage at the sub-national level.

**Supplementary Information:**

The online version contains supplementary material available at 10.1186/s12884-022-05080-5.

## Background

Reducing the maternal mortality ratio is a pivotal target as part of the ‘Sustainable Development Goal 3’, which affirms ‘to ensure healthy lives and promote wellbeing for all at all ages’ [1]. Maternal mortality is a crucial health indicator largely resulted from causes which are preventable and treatable [2]. Maternal health, which refers to the health status of a woman during the period of pregnancy, childbirth, and postpartum [3], has the potential of affecting significant maternal and child health outcomes, therefore the domain of maternal health assumes a pivotal role from the development perspective of a nation. Health system strengthening in terms of increased quality of care, health infrastructure, finance, and human resources can help bolster the maternal health service coverage, which in turn can have multiple positive implications on maternal, neonatal and child health statuses. The government of India has initiated many schemes and programmes to improve accessibility, affordability and quality of maternal health service coverage across the country [4–6]. Reaffirming its commitment towards achieving better maternal health outcomes, the Indian government, under the National Health Mission (NHM), introduced a novel strategic approach for ‘Reproductive, Maternal, Newborn, Child and Adolescent Health (RMNCH + A)’ [7]. This ‘continuum of care’ of RMNCH + A refers to an integrated health service accessible to women, neonatal, infants or adolescents over the dimension of time (before pregnancy, during pregnancy, childbirth and postnatal) and place (family and community care, outpatient and outreach services and hospital and health facilities) [8]. Maternal health thus, is one of the key thematic areas under the strategy of RMNCH + A, which aims to promote linkages between diverse health interventions to reduce maternal, neonatal and child mortality rates.

According to a guideline by the Indian Ministry of Health and Family Welfare (MoHFW), based on the time dimension, the health care services for a pregnant woman are classified mainly into three types, namely; antenatal/ prenatal care, intranatal/intrapartum care, and postnatal/postpartum care [3]. Antenatal care (ANC), which refers to the systemic care and supervision recommended for women during their pregnancy, is largely a platform for preventive and promotive health care services. ANC primarily aims to monitor the progress of fetal growth and wellbeing of the mother and fetus, thereby facilitating the timely management of obstetric complications. MoHFW guidelines recommend a minimum of four ANC visits in which the ANC registration and first ANC checkup within the first trimester (within 12 weeks) are accorded a high priority [3]. The World Health Organization (WHO) recommends various interventions ranging from dietary counselling, nutrient supplementation, and essential screening tests to improve the utilization and quality of antenatal care [9]. Another important stage of care in the continuum of RMNCH + A is intranatal/ intrapartum care (INC), which refers to systemic care for a pregnant woman during labour and delivery [3]. The time of birth is considered extremely crucial for both mother and the child as the chances of complications are high during this stage [10]. Sufficient literary evidence supports the prevalence of high-risk pregnancy and pre-term birth among the Indian and other south Asian populations [11–15]. Therefore, the delivery of the baby crucially requires an appropriate setting, where lifesaving equipment is available and hygienic conditions are fully maintained. Utilization of public, private or charitable trust/Non-Governmental Organization health facilities during labour and childbirth, referred to as Institutional delivery, is a significant means of timely management of obstetric complications [16]. However, literature reported that a considerable proportion still deliver at home in India attributed to social, cultural, physical and financial factors [17]. Increased skilled birth attendant (SBA) coverage is associated with a reduced probability of maternal deaths [16], especially for those women who prefer to deliver at home in India. The third stage of care is the postnatal/postpartum care (PNC), which involves systemic care of women after delivery, where the first 42 days (6 weeks) are conventionally treated as the postpartum period [3]. MoHFW guideline recommends at least three postnatal visits to the health facility after an uncomplicated normal institutional delivery in India. Evidence indicates that a larger proportion of neonatal deaths occurs in the first week of postnatal, with the highest number in the first three days [18].

To date, limited literature exists on the measurement of maternal health service coverage at the sub-national level in India. Prior research mainly focused on the theme of Reproductive, Maternal, Newborn and Child Health (RMNCH) service coverage and their inequities at national and sub-national levels in India. The majority of them used Coverage Gap Index (CGI) as an outcome measure of the RMNCH service coverage, which is primarily constituted by the domains of reproductive services, maternal and newborn care, immunization and management of illness [19–21]. However, there were only two indicators, namely ‘skilled birth attendant’ and ‘antenatal care coverage’ under the domain of maternal and newborn care, which makes the measurement of maternal health service coverage narrow and incomplete. To the best of our knowledge, no published study has estimated and examined maternal health service coverage using a composite index. The present study addresses this lacuna by constructing a comprehensive ‘Maternal Health Service coverage Index’ (MHSI), which includes a cumulative set of relatively large maternal health indicators categorized into three domains of antenatal, intranatal and postnatal care coverage, which is a major highlight of the study. The use of a composite index value would help us estimate the maternal health service coverage for a particular country/ state and compare them. The MHSI, as a summary measure, will reduce the reporting burden and help in expediting the systematic monitoring of maternal health service coverage in large entities like states and countries. Further, the constructed MHSI has been used for the identification of the low, medium and high coverage regions across different time series points in India, a theme which was largely unexplored in the previous studies. This shall help states identify lagged areas, introduce targeted interventions and prioritize the areas (antenatal, intranatal and postnatal care) to be intervened. Therefore, in this backdrop, utilising a nationally representative data, the specific objectives of the study are outlined as; a) to comprehensively measure maternal health service coverage at the sub-national level in India by constructing a composite index using a rigorous methodology; b) to map and categorize India into low, medium and high maternal health coverage zones; c) to examine the sub- national areas (states and union territories of India) which have transitioned from low to higher category of coverage and vice versa across the different time points.

## Methods

### Data source

The study extracted secondary data for three financial years (2017–18 to 2019–20) on various health indicators of maternal health service coverage from the Health Management Information System (HMIS) portal, an initiative under the National Health Mission (NHM) by the Government of India. HMIS is free of cost, publicly available web-based routine monitoring system of the vital Reproductive Maternal Newborn Child Health (RMNCH) data uploaded by the States and Union Territories (UT) in India. HMIS portal is the only data source that provides time-series data on a number of indicators of the Reproductive Maternal Newborn Child Health (RMNCH) domain in India, which is an added advantage as it allows undertaking temporal analysis and over time incremental changes. The HMIS portal that provides annual data on a financial year basis has been reporting detailed data at the sub-national level since 2017. A total of 29 states and 5 UTs have been included in examining maternal health service coverage at the sub-national level in India during the period 2017–18 (base year) and 2019–20 (reference year). The Indian states and union territories under the study have also been classified into four geographical zones, namely north, west, east & north east and south [22]. The raw and coded data has also been uploaded to Mendeley data repository [23] to provide free and unrestricted access to this time series data for facilitating transparency and reproducibility of the results.

### Selection and classification of indicators

The key HMIS indicators at the sub-national level are segregated into different domains, namely child health, family planning, maternal health and the ‘other’ miscellaneous. Since the present study aimed to construct a composite index for maternal health service coverage at the state and UT levels, the individual indicators have been selected from the domain of maternal health based on the opinion of medical experts and the public health professionals along with an extensive review of the literature. The experts mainly considered the relevance, usefulness, importance, and distinctiveness of the indicators in the valuation of the maternal health service coverage. In consensus with the experts, the study finalized 15 individual indicators to compute the Maternal Health Service Coverage Index (MHSI). It is to be noted that because of the difference in population composition and territory of Indian states, it was not appropriate to choose the indicators reported in the absolute values for the construction of maternal health service coverage. Hence, the study considered the relative measure of the selected indicators for the purpose of index construction.

The selected indicators were further classified under 3 domains of Antenatal care (ANC) coverage, Intranatal care (INC) Coverage and Postnatal care (PNC) coverage based on various guidelines by WHO and the Ministry of Health and Family Welfare [3, 9, 10, 18]. Furthermore, the study classified the component indicators of the ANC coverage domain into 3 sub-domains, namely ‘ANC registration & checkups (ANC1)’, ‘Supplementation & prophylaxis in pregnancy (ANC2)’, and ‘Essential screening tests (ANC3)’. It is to be noted that though all the women do not need c-section delivery, we included c-section delivery as a vital indicator under the domain of INC coverage. This was in view of considering c-section delivery as an important maternal health service indicator, which reflects the very availability of maternal health services, and may contribute to the maternal health service coverage. For instance, if a woman is in need of c-section delivery, but there is no availability of specialist or related facility due to certain reason, in such cases indicator of c-section delivery may become a service coverage indicator. Moreover, with increasing marriageable age [24] and the rising trend of the high-risk pregnancy in India [15], coupled with evolving literature on the need for accounting maternal choices in the type of delivery, have increased the demand for c-section delivery in India [25, 26], thereby making it an emerging service indicator for the maternal health. Therefore, in the light of changing times and dynamic health needs, this is another reason for including ‘c-section delivery’ as an indicator in our study and is one of the novelties of the study. A detailed description of the maternal health service coverage indicators has been given in Table 1.

**Table 1:** Operational definitions of indicators for the Maternal Health Service Coverage Index (MHSI)

Domains/Sub-domains/Indicators	Definition	Numerator	Denominator
**Domain I: *Antenatal Care (ANC) Coverage***			
**ANC sub-domain I:***ANC Registration & checkup (ANC1)*			
1. ANC Registration ***(ANC1_1)***	Percentageof first Trimester registration to total ANC Registrations	The number of pregnant women registered for ANC within the first trimester	The total number of pregnant women registered for ANC
2. ANC Check-ups ***(ANC1_2)***	Percentage Pregnant Woman received 4 ANC checkups to total ANC Registrations	The number of Pregnant Woman received 4 ANC checkups	The total number of pregnant women registered for ANC
**ANC sub-domain II:***Supplementation & prophylaxis in pregnancy (ANC2)*			
3. Immunization against Tetanus ***(ANC2_1)***	Percentage of pregnant women received TT2 + TT Booster to total ANC Registration	The number of pregnant women who received TT2 + TT Booster	The total number of pregnant women registered for ANC
4. IFA Supplementation ***(ANC2_2)***	Percentage of pregnant women were given 180 IFA tablets to total ANC Registration	The number of pregnant women given 180 IFA tablets	The total number of pregnant women registered for ANC
5. Anaemia treatment coverage ***(ANC2_3)***	Percentage of pregnant women with severe Anaemia (Hb < 7) treated at an institution to total ANC Registration	The number of pregnant women with severe Anaemia (Hb < 7) treated at an institution	The total number of pregnant women registered for ANC
6. Calcium supplementation ***(ANC2_4)***	Percentage of pregnant women were given 360 Calcium tablets to total ANC Registration	The number of pregnant women were given 360 Calcium tablets	The total number of pregnant women registered for ANC
7. Deworming ***(ANC2_5)***	Percentage of pregnant women were given one Albendazole tablet after 1st trimester to total ANC Registration	The number of pregnant women were given one Albendazole tablet after 1st trimester	The total number of pregnant women registered for ANC
**ANC sub-domain III:***Essential screening tests (ANC3)*			
8. Screening for Diabetes ***(ANC3_1)***	Percentage of pregnant women tested for blood sugar using OGTT to Total ANC Registration	The number of Pregnant women tested for blood sugar using OGTT	The total number of pregnant women registered for ANC
9. Screening for Syphilis ***(ANC3_2)***	Percentage of pregnant women tested for Syphilis to Total ANC Registration	The number of Pregnant women tested for Syphilis	The total number of pregnant women registered for ANC
10. Screening for HIV ***(ANC3_3)***	Percentage of females screened for HIV to total ANC Registration	The number of females screened for HIV	The total number of pregnant women registered for ANC
**Domain II: *Intranatal care (INC) coverage***			
11. SBA attended home deliveries ***(INC_1)***	Percentage of SBA attended home deliveries to total reported home deliveries	The number of home deliveries attended by SBA trained (Doctor/ Nurse/ ANM)	The total number of home deliveries reported
12. Institutional delivery ***(INC_2)***	Percentage of institutional deliveries to total reported deliveries	The number of deliveries conducted in both public and private institutions	The total number of reported deliveries
13. C-Section deliveries ***(INC_3)***	Percentage of c-section deliveries to reported institutional deliveries	The number of c-section deliveries conducted in both public and private institutions	The total number of reported institutional deliveries
14. Treatment for obstetric complications ***(INC_4)***	Percentage cases of pregnant women with obstetric complications and attended to reported deliveries	The number of Pregnant women with obstetric complications and attended	The total number of reported deliveries
**Domain III: *Postnatal care (PNC) coverage***			
15. Postpartum checkup ***(PNC_1)***	Percentage of women getting 1st postpartum checkup between 48 h and 14 days to total reported deliveries	The number of women getting 1st postpartum checkup between 48 h and 14 days	The total number of reported deliveries

### Data processing

Five percent of the obtained data on the selected 15 indicators of maternal health service coverage were randomly chosen and cross-checked/rechecked for the errors in the data entry to make sure that there was no error in the data entry while copying/transferring the data from the HMIS web portal [27]. Followed by that, the data was checked for the missing values with regard to the identified 15 MHSI indicators. Due to the availability of data for 3 financial years only, the standard imputation techniques and extrapolation and interpolation techniques could not be applied to treat the missing values [28]. The states or UTs with missing values for more than one specific year on any indicator have been excluded from the study. As per this criterion, Daman & Diu and Lakshadweep were removed from the study. The states/UTs having missing values only for one particular year were replaced by the available values of the most recent year [29].

### Computation of Maternal Health Service-coverage Index (MHSI)

#### Data normalization

The extracted data on the selected Maternal Health Service coverage Index (MHSI) from the HMIS were subjected to normalization based on the ‘Minimum–Maximum approach’ used by the United Nations Development Programme (UNDP) for the computation of Human Development Index (HDI) values. Each of the selected 15 indicators is positive in nature, where the resultant higher index value shall represent the positive result (higher the value, better the maternal health service coverage). Owing to the “higher the better” nature of the individual values, the said data was normalised using the following formula [30]:

$${N}_{i}=\frac{({A}_{i}-{L}_{i})}{({U}_{i}-{L}_{i})}$$

where *N*_*i*_ is the normalised value of the *i*^*th*^ indicator, *A*_*i*_ represents the Actual value of the *i*^*th*^ indicator, and *U*_*i*_ and *L*_*i*_ denote the maximum and minimum values (goal posts) of the dimension indicators, respectively. The UNDP, in its reports [31], recommended the use of absolute goal posts over the observed goal posts in the normalization process. The study fixed 100% (aspirational goal) and 0% (natural zero) as the maximum and minimum values (goal posts), respectively, from which the individual indicators were standardised.

#### Construction of MHSI: different scenarios

Followed by the calculation of normalized individual indicators, the Maternal Health Service Coverage Index (MHSI) involves three detailed steps. The first step was to estimate three sub-indices of the ANC, i.e., ANC1, ANC2, and ANC3, and to estimate separate indices of INC and PNC. Since both domains of INC and PNC coverage have not been segregated further, the study directly computed INC and PNC index from its individual indicators. Followed by step 1, the study computed the ANC index from its three subindices. Finally, in step 3, the study aggregated ANC, INC, and PNC indices to compute a comprehensive cumulative index for ‘Maternal Health Service Coverage’.

**Fig. 1:**
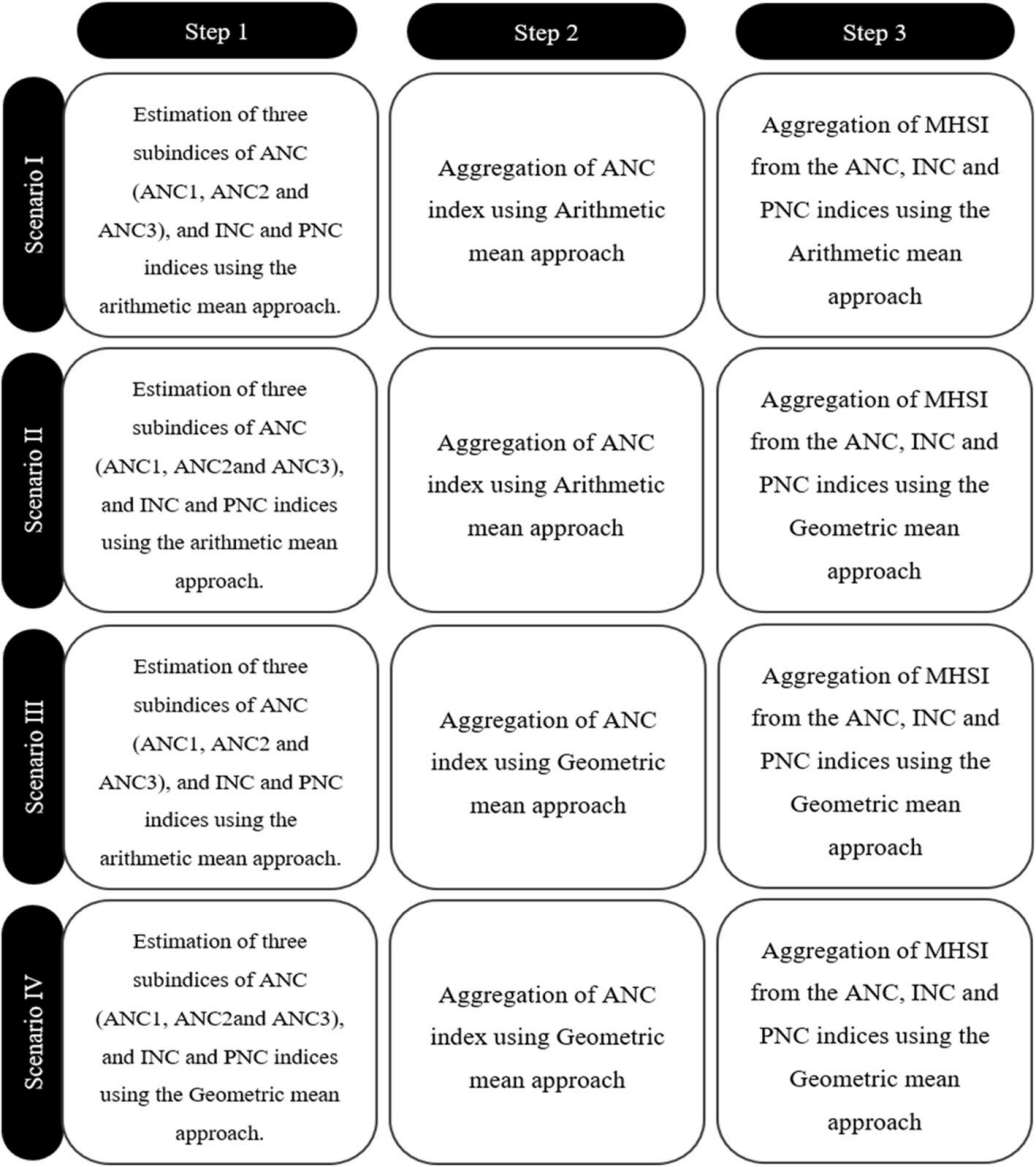
Different scenarios of the aggregation of domains and subdomains of MHSI

The study employed four different scenarios using the aforementioned three steps for the computation of MHSI. The different scenarios have been explained in detail in Fig. 1. Further, an additional file 5 clearly explains the formula used in each step of four different scenarios dealt in the study. Scenarios differ by choice of aggregation method, i.e., usage of arithmetic mean approach and geometric mean approach [31], which is primarily based on the procedure followed for the construction of the Human Development Index (HDI) by the United Nations Development Programme (UNDP). In scenario I, all the sub-indices and domain indices of MHSI were aggregated through the arithmetic mean approach. It is a basic and popular method of aggregating the indicators. However, the major disadvantage is that the results are largely affected by the extreme values and are not robust in nature [31]. However, the usage of arithmetic mean is more suitable in those cases where the data does not contain outliers or skewed data and normalization is carried from the observed values of the data distribution [31, 32]. In scenarios II and III, we used both arithmetic mean and geometric mean approaches, as detailed in Fig. 1 and Additional file 5. Since these two scenarios also incorporate the use of the arithmetic mean at different stages, it is also plagued with similar problems as highlighted above (less robust for policy formulations). In contrast to scenario I, scenario IV aggregated all the sub-indices and domain indices of MHSI through the geometric mean approach. The geometric mean approach has an advantage over the arithmetic mean approach (used in the scenario I) as it is less affected by skewed data and extreme values, thereby integrating the overall balance in the data distribution [31]. It is considered to be a robust and higher-level approach as per the UNDP methodology for constructing a composite index, therefore more suitable for decision making and policy interventions. Microsoft Office Excel 2019 and IBM SPSS version 24 were employed for the construction of the index and computation of related descriptive statistical measures.

#### Categorization and mapping of Indian states and Union Territories

The index values of the MHSI ranges between 0 and 1, where values close to 1 indicate high maternal service coverage and value close to zero correspond to low coverage. Furthermore, the study categorized Indian states into low, medium, and high categories based on the MHSI score. The study considered the period 2017–2018 as the base year and calculated the 33^rd^ and 66^th^ percentile scores of the MHSI values of Indian states. These percentile values were used as the criteria for determining the category Indian state belonged to. The Indian states with an MHSI value below the 33^rd^ percentile were categorised to the low category, those with above 66^th^ percentile were categorised to the high category, and the ones having a score between 33^rd^ and 66^th^ percentile were assigned to the medium category. The study categorised Indian states based on MHSI values in all four scenarios. The details of the 33^rd^ and 66^th^ percentile scores in different scenarios have been described in Additional file 1. Furthermore, the study geospatially mapped the Indian states into different MHSI zones using the Arch GIS 10.8 software.

## Results

### Descriptive characteristics of maternal health service coverage indicators

**Fig. 2:**
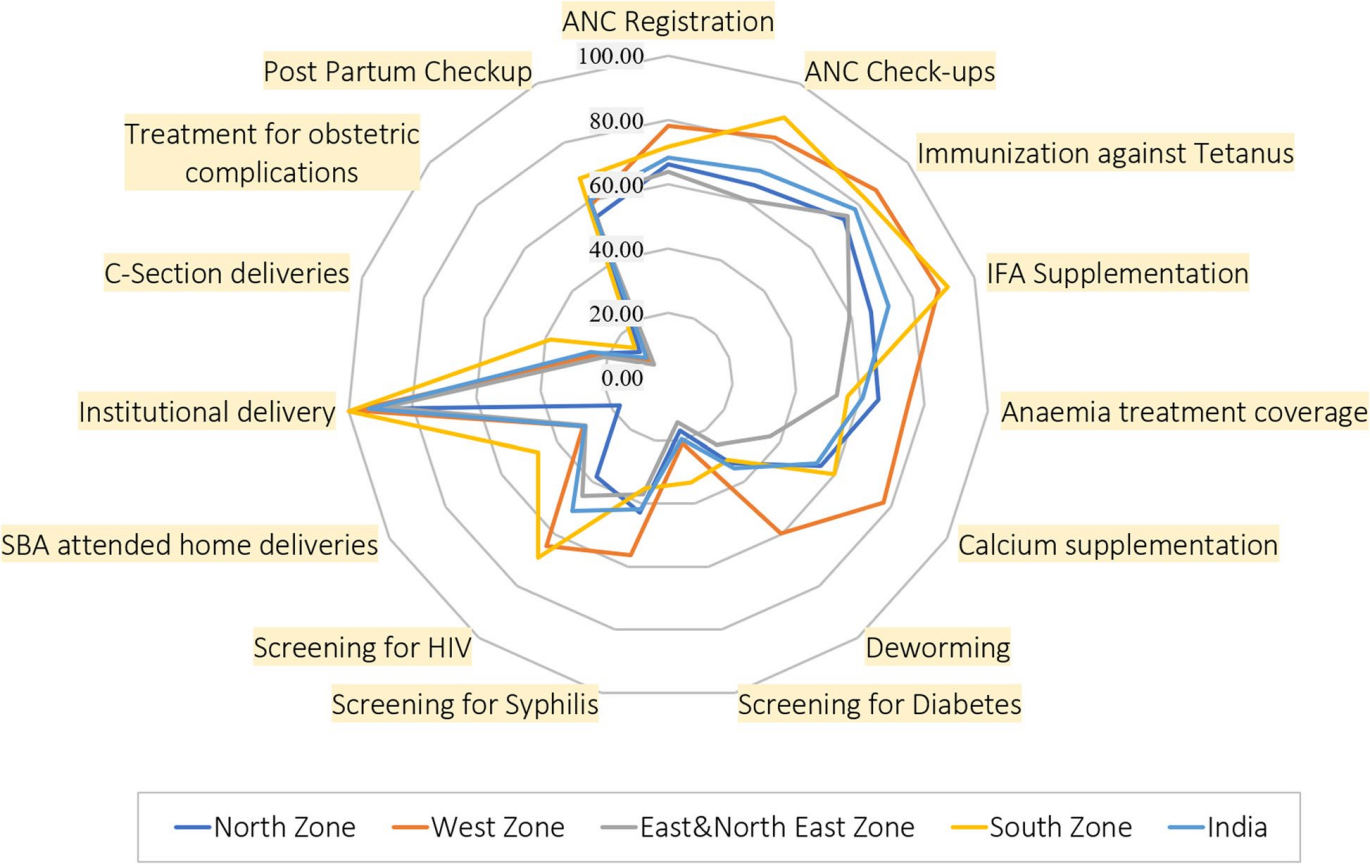
Radar Diagram exhibiting the extent of Maternal health service coverage in India across different zones over the period 2017–2020

Table 2 and Fig. 2 describe the characteristics of selected maternal health service coverage indicators at the regional and national levels. Out of the 15 indicators, institutional delivery showed the highest mean percentage across India and every region. The study observed that, on an average, 93.7% of the total reported delivery delivered at the institution (both public and private) in India. Across India, ‘treatment for obstetric complications’ was observed to be the lowest (9.3%) among all the indicators.

**Table 2:** Descriptive characteristics of indicators of maternal health service coverage at regional and national level over a span of three years (2017–18 to 2019–20)

Indicators	Mean (%)					Coefficient of variation (%)				
	North zone	West zone	East & north east zone	South zone	**India**	North zone	West zone	East & north east zone	South zone	**India**
**Domain I: *Antenatal Care Coverage (ANC)***										
**ANC Sub-domain I:***ANC Registration & checkup (ANC1)*										
ANC Registration	66.3	78.1	64	71.7	68.5	21.7	18.1	30.7	29.9	26.8
ANC Check-ups	65.5	81.6	60.3	88.4	70.4	27.5	10	33.7	17.9	29.1
**ANC Sub-domain II:***Supplementation & prophylaxis in pregnancy (ANC2)*										
Immunization against Tetanus	73.6	86.9	75	82.7	78.1	27.5	21.8	22.3	35.9	27
IFA Supplementation	66.2	88.4	59.3	91.3	71.9	28.6	12.1	40	28	34.6
Anaemia treatment coverage	65.7	75	52.6	56	60.7	22.8	21.9	39.4	33.1	32.8
Calcium supplementation	54.9	77.3	36.5	59.7	53.2	41.4	17.2	59.9	55.9	50.7
Deworming	32.9	59.7	25.8	31.7	34.7	63.8	33.2	79.8	69.4	68.5
**ANC Sub-domain III:***Essential screening tests (ANC3)*										
Screening for Diabetes	16.9	20.7	14.1	33.2	19.4	97.2	120.5	151.5	135.9	140
Screening for Syphilis	42.9	56.4	37	35.2	41.6	47.6	92.8	59.5	53.6	70.6
Screening for HIV	37.9	64.6	45.4	69.1	51	58.6	81.3	81.8	48.5	74.7
**Domain II: *Intranatal care coverage (INC)***										
SBA attended home deliveries	17.3	30.5	29.6	46.6	30.1	80.3	64.5	67.5	50.2	69.7
Institutional delivery	93.8	98.3	88.6	99.8	93.7	5.2	2.1	12.2	0.3	9
C-Section deliveries	24	23	21	38.6	25.3	48.9	47.9	44.9	18.2	46.5
Treatment for obstetric complications	11.8	7.6	6.1	13.9	9.3	79.8	43.7	69.2	121.2	101.9
**Domain III: *Postnatal care coverage (PNC)***										
Postpartum Check-up	54.7	59	61.2	67.6	60.2	53.21	37.4	36.9	65.3	48.1

In most of the indicators, the performance of the south zone and west zone were notable. Of the 4 zones, the south zone recorded highest mean percentage coverage for ANC check-ups (88.4%), IFA supplementation (91.3%), institutional deliveries (99.8%), screening for diabetes (33.2%), screening for HIV (69.1%), SBA attended home deliveries (46.6%), c-section deliveries (38.6%), postpartum check-up (67.6%) and treatment for obstetric complications (13.9%). In contrast, the west zone registered the highest mean percentage coverage for ANC registration (78.1%), immunization against tetanus (86.9%), anemia treatment coverage (75%), calcium supplementation (77.3%), deworming (59.7%) and screening for syphilis (56.4%). East & north east zone registered the lowest mean percentage coverage in 10 out of 15 indicators namely, ANC registration (64%), ANC check-up (60.3%), IFA supplementation (59.3%), anemia treatment coverage (52.6%), calcium supplementation (36.5%), deworming (25.8%), screening for diabetes (14.1%), institutional delivery (88.6%), c-section delivery (21%) and treatment for obstetric complications (6.1%).

On the other hand, the north zone registered the lowest mean percentage coverage for immunization against tetanus (73.6%), screening for HIV (37.9%), SBA attended home deliveries (17.3%) and postpartum checkup (54.7%). Among the indicators, none of the zones recorded more than 50% coverage in screening for diabetes, SBA attended home deliveries, c-section deliveries and treatment for obstetric complication. Furthermore, estimates of the coefficient of variation showed indicator of ‘institutional delivery’ has the lowest and the indicator of ‘the screening for diabetes’ has the highest variations in India.

### Composite Maternal Health Service Coverage Index (MHSI) and its comparison between the base year and reference year

The study computed MHSI using different methodological based scenarios already explained in Fig. 1. The MHSI values based on four different approaches for the base year (2017–18) and reference year (2019–20) have been presented in the Additional file 2. In scenario I, all the sub-indices and domain indices of MHSI were aggregated through the arithmetic mean approach, whereas, in Scenario IV, the geometric mean approach was employed [31]. For conciseness, MHSI values across the base year and reference year were compared only for scenarios I (Table 3) and IV (Table 4). However, in the interest of the large audience, details of MHSI values across the base and reference years for scenarios II and III have also been presented in Additional files 3 and 4. Nevertheless, these tables do not form the part of analytic inferences of the present study.

**Table 3:** Incremental change of MHSI values and categorization of states in the base year and reference year (***Scenario I***)

State/UTs	MHSI Values in base year (2017–18)		MHSI Values in reference year (2019–20)		Incremental Changes in MHSI	
	**Index value**	**Category**	**Index value**	**Category**	**Change in value**	**Change in category**
**North zone**	**0.462**	**Medium**	**0.502**	**Medium**	**0.04**	** → **
Chandigarh^a^	0.370	Low	0.470	Medium	0.100	**↑**
Delhi^a^	0.437	Low	0.439	Medium	0.002	**↑**
Haryana	0.549	Medium	0.530	Medium	-0.019	** ← **
Himachal Pradesh	0.608	High	0.699	High	0.090	** → **
Jammu& Kashmir	0.505	Medium	0.499	Medium	-0.006	** ← **
Punjab	0.645	High	0.663	High	0.018	** → **
Rajasthan	0.309	Low	0.345	Low	0.036	** → **
Uttar Pradesh	0.318	Low	0.436	Low	0.119	** → **
Uttarakhand	0.419	Low	0.433	Low	0.015	** → **
**West zone**	**0.530**	**Medium**	**0.579**	**High**	**0.049**	**↑**
Chhattisgarh	0.597	High	0.572	High	-0.025	** ← **
Dadra & Nagar Haveli^a^	0.556	Medium	0.663	High	0.107	**↑**
Goa	0.564	Medium	0.660	High	0.096	**↑**
Gujarat	0.568	High	0.654	High	0.086	** → **
Madhya Pradesh	0.356	Low	0.337	Low	-0.019	** ← **
Maharashtra	0.540	Medium	0.588	High	0.048	**↑**
**East & north east zone**	**0.478**	**Medium**	**0.491**	**Medium**	**0.013**	** → **
Andaman & Nicobar Island^a^	0.644	High	0.669	High	0.025	** → **
Arunachal Pradesh	0.378	Low	0.337	Low	-0.042	** ← **
Assam	0.533	Medium	0.626	High	0.093	**↑**
Bihar	0.334	Low	0.369	Low	0.035	** → **
Jharkhand	0.410	Low	0.467	Medium	0.057	**↑**
Manipur	0.438	Low	0.426	Low	-0.012	** ← **
Meghalaya	0.340	Low	0.340	Low	0.000	** → **
Mizoram	0.481	Medium	0.346	Low	-0.135	**↓**
Nagaland	0.371	Low	0.372	Low	0.001	** → **
Odisha	0.591	High	0.692	High	0.101	** → **
Sikkim	0.629	High	0.628	High	-0.001	** ← **
Tripura	0.479	Medium	0.504	Medium	0.025	** → **
West Bengal	0.584	High	0.614	High	0.030	** → **
**South zone**	**0.620**	**High**	**0.578**	**High**	**-0.042**	** ← **
Andhra Pradesh	0.605	High	0.660	High	0.055	** → **
Karnataka	0.712	High	0.723	High	0.012	** → **
Kerala	0.858	High	0.748	High	-0.110	** ← **
Puducherry^a^	0.546	Medium	0.388	Low	-0.158	**↓**
Tamil Nadu	0.462	Medium	0.476	Medium	0.015	** → **
Telangana	0.540	Medium	0.472	Medium	-0.068	** ← **
**India**	**0.523**	**Medium**	**0.538**	**Medium**	**0.015**	** → **

**↑** Upward transition from a lower category to higher; → No change in category, but MHSI value increased; → No change in category but MHSI value decreased; **↓** Downward transition from a higher category to lower

^a^Denotes Union Territory (UT)

**Table 4:** Incremental change of MHSI values and categorization of states in base year and reference year (***Scenario IV***)

State/UTs	MHSI Values in Base Year (2017–18)		MHSI Values in Reference year (2019–20)		Incremental Changes in MHSI	
	**Index value**	**Category**	**Index value**	**Category**	**Change in value**	**change in category**
**North zone**	**0.315**	**Medium**	**0.362**	**Medium**	**0.046**	** → **
Chandigarh^a^	0.183	Low	0.331	Medium	0.148	**↑**
Delhi^a^	0.332	Medium	0.312	Medium	-0.020	** ← **
Haryana	0.404	Medium	0.398	Medium	-0.006	** ← **
Himachal Pradesh	0.483	High	0.575	High	0.092	** → **
Jammu& Kashmir	0.342	Medium	0.376	Medium	0.034	** → **
Punjab	0.504	High	0.545	High	0.041	** → **
Rajasthan	0.234	Low	0.224	Low	-0.009	** ← **
Uttar Pradesh	0.211	Low	0.316	Medium	0.105	**↑**
Uttarakhand	0.307	Medium	0.311	Medium	0.005	** → **
**West zone**	**0.393**	**Medium**	**0.427**	**High**	**0.034**	**↑**
Chhattisgarh	0.504	High	0.469	High	-0.035	** ← **
Dadra & Nagar Haveli^a^	0.412	Medium	0.477	High	0.065	**↑**
Goa	0.430	High	0.555	High	0.125	** → **
Gujarat	0.402	Medium	0.490	High	0.088	**↑**
Madhya Pradesh	0.241	Low	0.207	Low	-0.034	** ← **
Maharashtra	0.422	Medium	0.477	High	0.055	**↑**
**East& north east zone**	**0.324**	**Medium**	**0.337**	**Medium**	**0.013**	** → **
Andaman & Nicobar Island^a^	0.528	High	0.532	High	0.004	** → **
Arunachal Pradesh	0.289	Low	0.252	Low	-0.036	** ← **
Assam	0.384	Medium	0.478	High	0.094	**↑**
Bihar	0.201	Low	0.231	Low	0.030	** → **
Jharkhand	0.277	Low	0.332	Medium	0.055	**↑**
Manipur	0.310	Medium	0.305	Medium	-0.005	** ← **
Meghalaya	0.258	Low	0.266	Low	0.008	** → **
Mizoram	0.321	Medium	0.248	Low	-0.073	**↓**
Nagaland	0.234	Low	0.247	Low	0.013	** → **
Odisha	0.456	High	0.570	High	0.114	** → **
Sikkim	0.488	High	0.492	High	0.004	** → **
Tripura	0.263	Low	0.269	Low	0.006	** → **
West Bengal	0.383	Medium	0.394	Medium	0.011	** → **
**South zone**	**0.408**	**Medium**	**0.389**	**Medium**	**-0.019**	** ← **
Andhra Pradesh	0.454	High	0.516	High	0.062	** → **
Karnataka	0.567	High	0.596	High	0.029	** → **
Kerala	0.545	High	0.529	High	-0.015	** ← **
Puducherry^a^	0.466	High	0.331	Medium	-0.135	**↓**
Tamil Nadu	0.180	Low	0.189	Low	0.009	** → **
Telangana	0.392	Medium	0.341	Medium	-0.051	** ← **
**India**	**0.358**	**Medium**	**0.377**	**Medium**	**0.019**	** → **

**↑** Upward transition from a lower category to higher; → No change in category but MHSI value increased; → No change in category but MHSI value decreased; **↓** Downward transition from a higher category to lower

^a^Denotes Union Territory (UT)

For scenario I (Table 3), the MHSI of India increased slightly from the base year (0.523) to the reference year (0.538), however, both values corresponded to medium level of maternal health service coverage. At the regional level, the MHSI value of west zone showed the highest incremental change of about 0.049 index points, which transitioned it from the category of medium MHSI in the base year to the high MHSI level in the reference year. In contrast, there is a negative change in the value of MHSI (-0.042) of the south zone, but it still remained under the category of high MHSI value. Overall, the state of Kerala (South zone) topped both in the base and reference year with MHSI scores of 0.858 and 0.748, respectively. Rajasthan (North zone) with an MHSI score of 0.309 and Arunachal Pradesh (East & north east zone) with a score of 0.337 were in the last position for the base year and reference year, respectively. The states/UTs, namely Dadra & Nagar Haveli (0.663), Goa (0.66) and Maharashtra (0.588) from the west zone and Assam (0.626) from the east zone transitioned upwards from medium to high category in the reference year, while others Chandigarh (0.470) and Delhi (0.439) from the north zone as well as Jharkhand (0.467) from east zone moved upwards from low to medium category. Only the states of Mizoram (0.346) of the east zone and Puducherry (0.388) of the south zone showed a downward transition, from medium to low MHSI category. The state of Uttar Pradesh (0.119) from the north zone and the UT of Dadra & Nagar Haveli (0.107) from the west zone showed the highest positive increment in MHSI value. While the highest negative decrement was recorded by the state of Mizoram (-0.135) and UT of Puducherry (-0.158).

For scenario IV (Table 4), which is based on the geometric mean approach, the MHSI value for India had marginally increased (by 0.019 index points) in the reference year (0.377) as compared to the base year (0.358). India has remained in the medium category, thereby indicating no upward or downward transition with respect to the maternal health service coverage. In terms of absolute index values, the highest increment in MHSI score (0.046 index points) was achieved by the north zone (0.362) in the reference year, however it remained in the medium category only. The west zone is the only region, which transitioned from the medium to high MHSI category in the reference year, and it recorded the highest MHSI value of 0.427, followed by the south zone (0.389). Apart from south zone, MHSI index scores increased for all other zones in the reference year. The study observed an upward transition of the MHSI category from low to medium in the following Indian states/UTs, namely Chandigarh (0.331) and Uttar Pradesh (0.316) from the north zone and Jharkhand (0.332) from the east & north east zone, while Dadra & Nagar Haveli (0.477), Gujarat (0.49) and Maharashtra (0.477) from the west zone and Assam (0.478) from east & north east zone showed an upward transition in category from medium to high. Only two states, Mizoram (0.248) from the east & north east zone and Puducherry (0.331) from the south zone showed a downward transition in the MHSI category. The state of Mizoram transitioned from medium to low category while that for the UT of Puducherry was from High to Medium category. The state of Goa (0.125) and UT of Chandigarh (0.148) recorded the highest positive incremental change in MHSI value, whereas the state of Mizoram (-0.073) and UT of Puducherry (-0.135) registered the highest negative decrement.

**Fig. 3:**
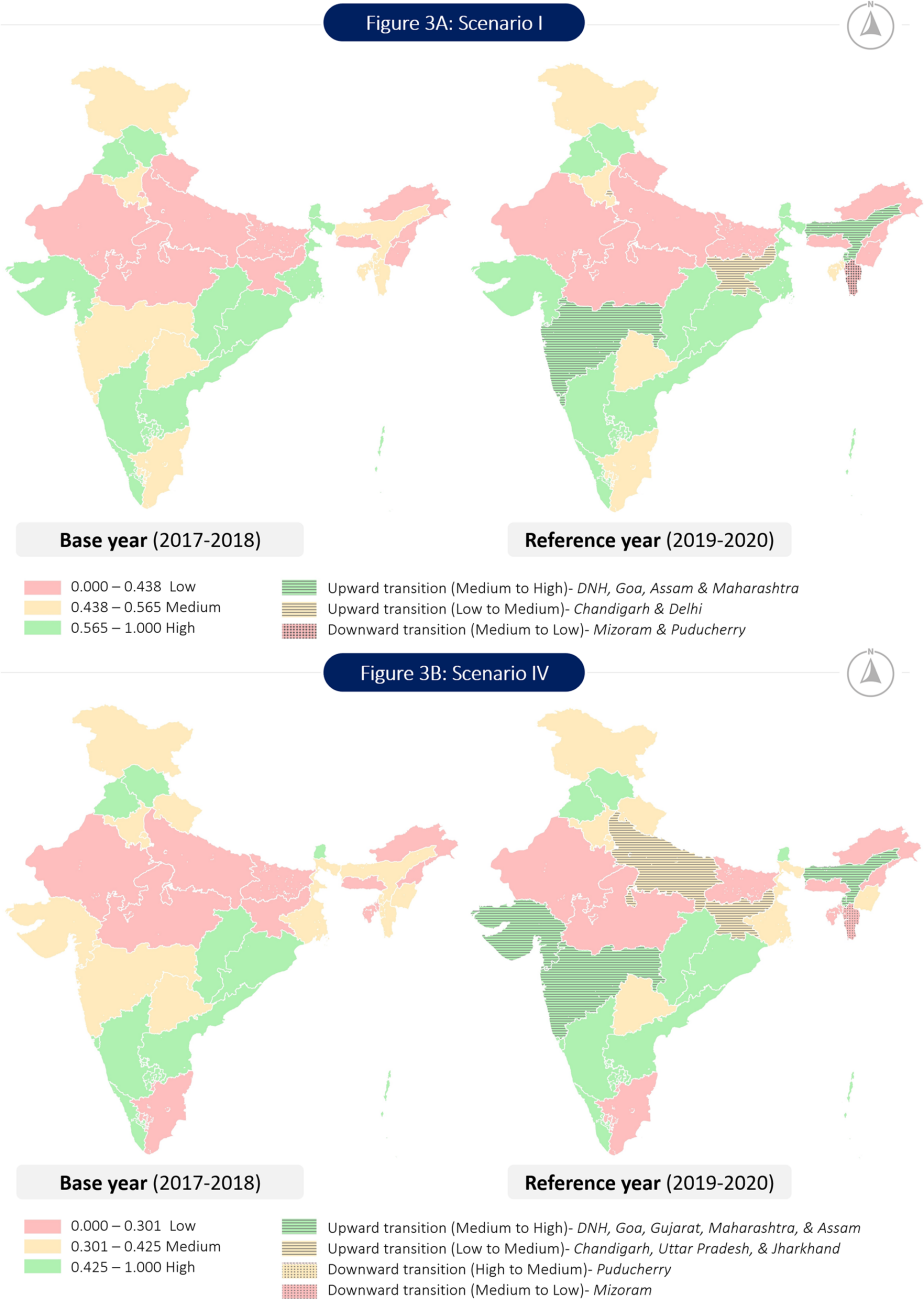
Extent of maternal health service coverage and transition of Indian states and Union territories for scenario I and scenario IV. *Source:* Author generated the map using Arc GIS 10.8

The transition of states across different MHSI categories based on the MHSI index values in the base year and reference year has also been presented through geo-spatial maps in Fig. 3 (Fig. 3A for scenario I and Fig. 3B for scenario IV).

## Discussion

Out of the 15 maternal health service indicators, institutional delivery (INC indicator), immunization against tetanus, ANC checkups and IFA supplementation (ANC indicators) recorded a relatively higher mean percentage (more than 70%) coverage at all India level for three years (2017–18 to 2019–20). Maternal health care services like institutional delivery and antenatal care coverage in India observed substantial progress in the past two decades with the implementation of plethora of targeted government driven programmes such as the National Health Mission (NHM), Maternity Benefit programmes like Janani Suraksha Yojana(JSY), Pradhan Mantri Surakshit Matritva Abhiyan (PMSMA) and Pradhan Mantri Matru Vandana Yojana (PMMVY) to name a few [4, 5, 33]. Recently launched ‘Anemia Mukt Bharat’ programme has given prime importance to IFA supplementation for pregnant women, which is a highly probable reason for enhanced IFA coverage [34]. However, the national mean percentage coverage of some of the indicators of maternal health service coverage is quite perturbing, such as; screening for diabetes (ANC indicator), SBA attended home deliveries, c-section delivery and treatment for obstetric complications (all INC indicators) as they recorded relatively low mean percentage (around 30%) coverage across the country. Some state specific studies recorded prevalence of gestational diabetes ranged from 9.89% to 35% [35–39], which is quite alarming. In this regard, in compliance with the recommendations of WHO and the International Association of Diabetes and Pregnancy Study Group (IADPSG), India requires region specific efforts to expand its coverage of screening for diabetes among pregnant women [38]. Though the present study observed lower mean percentage coverage for c-section delivery across India, nevertheless the growth rate of c-section delivery in India has doubled during 2005–06 to 2015–16 [40]. Low socio-economic status, awareness of available schemes, distance to the health facility, absence of transport, and social cultural norms can be identified for preference of home delivery and related maternal complications in India [41–44].

Both southern and western zones of India have outperformed the other zones on the coverage of a number of maternal health indicators. South zone, particularly has performed sizable well with respect to institutional deliveries, IFA supplementation, ANC checkups and screening for HIV [45–48]. The states of Kerala, Tamil Nadu, Karnataka and Andhra Pradesh from the south zone are the front runners in the overall ‘Sustainable Development Goals’ (SDGs) with relatively high SDG index scores, which has remarkably influenced their coverage and receipt of health care services such as institutional care and antenatal care coverage [49]. Consistently high HIV prevalence districts were clustered mainly in the south zone of the country [50], therefore, it is expected to have high percentage coverage in screening for HIV as a preventive measure. Furthermore, most of the states from south zone are known for their remarkable performance in literacy rates, particularly in female literacy [51], which can be a possible reason for increased awareness of HIV screening among south zone population. West zone particularly has recorded a substantial high coverage for immunization against tetanus, ANC registration, calcium supplementation and anemia treatment coverage. Effective planning and management of resources, re-establishment of outreach services, community links, supportive supervision and monitoring are the possible facilitating factors of immunization services including tetanus [52]. State governments of Gujarat, Chhattisgarh and Madhya Pradesh from the west zone have collaborated with WHO for calcium supplementation to pregnant women [53] possibly the reason for high ‘calcium supplementation’ coverage in the west zone.

North zone recorded the lowest coverage for indicators particularly with respect to screening for HIV and SBA attended home deliveries. Low female literacy and rigid socio-cultural factors of the majority of states from the north zone may have resulted in low SBA attended home deliveries [51]. However, Uttar Pradesh and Rajasthan from the north zone are observed to be the states which require the highest need for ‘Prevention of Maternal to Child Transmission’ (PMTCT) of HIV [54] and therefore demands high coverage in screening for HIV among pregnant women. East & north east zone has underperformed on a number of indicators, specifically for screening pregnant women for diabetes, c-section delivery and treatment for obstetric complications. The low female literacy rate [51] and the high proportion of tribal population [55] are expected to account for low c-section delivery rates in this zone. Majority of states from the east & north east zone recorded a low prevalence of diabetes in a nationwide study [56], making it a likely cause for low diabetes screening coverage. Inaccessible regional terrain, poor transportation facilities, availability of prompt medical services and behavioural factors (tobacco and alcohol use) are significantly associated with a low treatment coverage for obstetric complications [57].

The methodology adopted in the present study for data normalization and construction of MHSI is primarily based on United Nations Development Programme (UNDP)’s procedure to compute Human Development Index (HDI) [58], which is highly rigorous and standardized. Further, the study generated MHSI scores based on the choice of arithmetic mean and geometric mean approach for aggregating the 15 indicators into one composite index. The geometric mean approach (used in scenario IV) has an advantage over the arithmetic mean approach (used in scenario I) as it is less affected by skewed data and extreme values, thereby integrating the overall balance in the data distribution [31]. However, the usage of arithmetic mean is more suitable in those cases where the data normalization is carried from the observed values of the data distribution [32]. Therefore, comparison of index values across these two different approach-based scenarios are redundant in nature. We clarify that the study elucidates the comparison of MHSI values across two time periods (base year and reference year) within a given scenario and not across the two scenarios. Construction of MHSI for these two particular scenarios, facilitates the identification of those common states/union territories/regions, which have transitioned form a lower to higher category of maternal health service coverage or vice versa. The earlier attempts to measure maternal health service coverage have been limited and narrow in their approach. Composite Coverage Index (CCI), which comprised essential health interventions of RMNCH continuum of care [59], included only two indicators i.e., ‘skilled birth attendant’ and ‘antenatal care coverage’ under the domain of maternal and newborn care. In contrast, the present study specifically emphasized on maternal health service coverage rather than considering the consolidated RMNCH coverage as studied in the previous literature [19–21, 59, 60].

Different states/UTs/zones in India have performed variedly as far as MHSI scores are concerned. The UTs of Dadra & Nagar Haveli and Chandigarh showed upward transition of the MHSI category in both scenarios (I and IV). Dadra & Nagar Haveli transitioned from medium to high category and that of Chandigarh showed low to medium category. This might be likely due to the concentrated efforts of respective governments and also partly because these UTs are the front runners or performers in specific SDGs related to poverty, health, quality education and economic growth [49]. The state of Maharashtra and Assam showed an upward transition in both scenarios from medium to high MHSI category, while Jharkhand showed upward transition from low to medium category. Assam and Jharkhand have remarkably improved its maternal health outcomes and have recently been accorded a position of ‘achiever’ in a health index released by the National Institution for Transforming India (NITI) Aayog, which is the Indian government’s apex think tank [61]. While, the state of Maharashtra is a frontrunner across various dimensions of NITI Aayog’s health index. On the other hand, the state of Mizoram and the UT of Puducherry displayed downward transition in both scenarios. This might be due to relatively less coverage on a number of indicators like IFA supplementation [62], diabetes screening [56, 63], ANC checkups and treatment of obstetric complications. Out of the 4 zones, only the west zone transitioned upwards in the reference year from the medium to high MHSI category. The state of Goa and Maharashtra and the UT of Dadra and Nagar Haveli from the west zone performed substantially well, which gave an upsurge in the MHSI scores of the west zone. None of the zones showed downward transition across two time points for both the methodological scenarios. Region/state/UT specific health interventions are required to be undertaken to target low coverage indicators of maternal health for bolstering their performance to achieve positive incremental changes in maternal health outcomes over time.

The present study faces some limitations. The present study is based on the HMIS data that allows undertaking temporal analysis and over time incremental changes, nevertheless some issues still remain in terms of data quality and therefore the results of the present study should be cautiously used. Consideration of private sector, which reports at a lower rate than the public sector to the health facility data like HMIS, may affect the numerators of the MHSI individual indicators especially for the states with a greater predominance of private sector. Therefore, readers have to be watchful while drawing and generalizing the conclusions. There might be inherent issues with some HMIS derived denominators used to calculate the coverage estimates. The denominators indicates those females who were registered for a maternal services for instance ‘Total number of pregnant women registered for ANC’. It excludes that population who have not been registered for the said maternal service or did not seek maternal health care elsewhere. Those women might be marginalized, more vulnerable and lesser health status. This can lead to selection bias depending on proportion of women registered for any service. The external validation of MHSI individual indicators from HMIS data with that of state specific data or NFHS data and alike should be undertaken for better generalizability, which forms the future scope of this study. Besides, due to limited data availability at the sub-national level, the analysis pertains to a short time span of three years (2017–18 to 2019–20), hence the incremental changes in the maternal health service coverage reflect only the preliminary estimates, therefore should not be used to draw inferences for long term policy purposes. More time series data is required to confirm and substantiate the inferences of these short time series-based results. This leaves a lot of scopes for future research to undertake longitudinal analysis concerning the theme of the present study. Though the composite ‘Maternal Health service Index’ computed in the study involves a set of 15 indicators, which is relatively larger than the previous studies, however; data on only one indicator (postpartum checkup) was available and classified under the domain of postnatal care, which makes this domain under-represented for constructing the index. The government of India has initiated a number of schemes especially for nutritional interventions of pregnant and lactating women [64, 65] for antenatal and postnatal care. In this light, besides postpartum checkup, which is a globally recognised indicator [66], data on more indicators like nutritional support, screening for psychological wellbeing after delivery [67] and alike should be disseminated on the government portal for better representation of the PNC domain, which is an important food for thought for a revised version of this novel index in future. Additionally, though the study included ‘c-section delivery’ as an important indicator under the domain of intranatal care; nevertheless, the states need to be cautious and should take into account the trend/ prevalence of high-risk pregnancy, obstetric complications and evolving concept of maternal choice/decision while taking a decision on the inclusion of particular indicators like c-section delivery as a coverage indicator as all the women are not in need c-section delivery. Although the study envisioned to examine each and every state as well as union territories, however the two union territories of Daman & Diu and Lakshadweep were excluded from the analysis as they both registered missing values for more than one specific year on a particular maternal health indicator. Nevertheless, the present study has some strong points and strengths to offer. The novel ‘Maternal Health Service Coverage Index’ envisaged and constructed in the present study is a pioneer attempt to comprehensively quantify the sub-national coverage of maternal health service in India by including within its ambit 15 indicators spread across each of the three domains of antenatal care, intranatal care and postnatal care. No published study has elucidated the measurement of maternal health service coverage in terms of the broad spectrum of antenatal, intranatal and postnatal care coverage till date in India, though these domains hold prime importance and are widely valued by the reputed international and national organizations [3, 9, 68]. There is wide scope of usage for this unique ‘composite index’ as it provides a concise and quantified information of complex and multifaceted facts representing maternal health service coverage, which makes it easy to communicate and report for the policy purpose [69] at the sub-national level in the Indian setting*.* This composite summary measure, will reduce the reporting burden and speed up the monitoring of maternal health service coverage at sub-national level in India.The study has further mapped and categorized India into different zones, thereby providing a snapshot of low, medium and high maternal health service coverage states/union territories and regions. This shall help states identify and prioritize the laggard areas (within antenatal, intranatal and postnatal care) to be intervened. Furthermore, the study by considering a nationally representing data published by the Indian government, has facilitated the comparison of the maternal health service coverage performance at the sub-national level across different time points, which has allowed to identify which states/union territories and regions have transitioned from lower to a higher maternal health service coverage category and vice versa.

## Conclusion

Measuring and mapping of maternal health service coverage at the sub-national level can have significant policy implications for a developing country like India, which is making rapid strides in healthcare sector transformation. A composite summary measure to reflect the coverage of maternal health services can momentously help India to identify low coverage maternal health service indicators and recognize lagged zones, thereby warranting the concentrated programmatic and clinical efforts to detect, examine and target specific characteristics of a particular state/UT/region that has a significant effect on maternal health service coverage. The accessibility, availability and utilization of quality driven maternal health services are key for survival and wellbeing of both mother and child. A regular longitudinal and extensive monitoring of the performance of states with regard to essential maternal health services shall induce the country towards achieving the SDG target of improved maternal and child health outcomes.

## Supplementary Information

**Additional file 1. **Details of 33rd percentile and 66th percentiles scores in different scenarios (Scenario I to IV).

**Additional file 2. **Maternal Health Service Coverage Index (MHSI) values at sub-national level in India based four different scenarios for base year (2017-2018) and reference year (2019-2020).

**Additional file 3. **Incremental change of MHSI values and categorization of states in base year and reference year (Scenario II).

**Additional file 4. **Incremental change of MHSI values and categorization of states in base year and reference year (Scenario III).

**Additional file 5. **Formula used in the construction of MHSI in four different scenarios.

## Data Availability

The dataset generated and/or analysed during the current study are available in the Mendeley data repository, 10.17632/ngtpyv4zws.1, https://data.mendeley.com/datasets/ngtpyv4zws/1.

## Abbreviations

ANC: Antenatal care
CCI: Composite Coverage Index
CGI: Coverage Gap Index
DNH: Dadra & Nagar Haveli
HDI: Human Development Index
HIV: Human Immunodeficiency Virus
HMIS: Health Management Information System
IADPSG: International Association of Diabetes and Pregnancy Study Group
IFA: Iron Folic Acid
INC: Intranatal care
JSY: Janani Suraksha Yojana
MHSI: Maternal Health Service coverage Index
MoHFW: Ministry of Health and Family Welfare
NHM: National Health Mission
NITI: National Institution for Transforming India
PMTCT: Prevention of Maternal to Child Transmission
PMMVY: Pradhan Mantri Matru Vandana Yojana
PMSMA: Pradhan Mantri Surakshit Matritva Abhiyan
PNC: Postnatal care
RMNCH: Reproductive, Maternal, New born and Child Health
RMNCH + A: Reproductive, Maternal, Newborn, Child and Adolescent Health
SBA: Skilled Birth Attendant
SDG: Sustainable Development Goals
UNDP: United Nations Development Programme
UT: Union Territories
WHO: World Health Organization
