# Supplementary material for: Measurement and mapping of maternal health service coverage through a novel composite index: a sub-national level analysis in India
Source: BMC Pregnancy Childbirth. 2022 Oct 10;22:761. doi: 10.1186/s12884-022-05080-5 (PMC9552458; doi:10.1186/s12884-022-05080-5)
Supplement: Supplementary file 1 — Additional file 1. Details of 33rd percentile and 66th percentiles scores in different scenarios (Scenario I to IV). [file 12884_2022_5080_MOESM1_ESM.pdf]

Additional file 1

Additional file 1.pdf

Title: Details of 33rd percentile and 66th percentiles scores in differnet scenarios (Scenario I to IV)

	**Scenario 1**	**Scenario II**	**Scenario III**	**Scenario IV**
33rd Percentile	0.438	0.415	0.407	0.301
66th percentile	0.565	0.544	0.520	0.425
