# Supplementary material for: Measurement and mapping of maternal health service coverage through a novel composite index: a sub-national level analysis in India
Source: BMC Pregnancy Childbirth. 2022 Oct 10;22:761. doi: 10.1186/s12884-022-05080-5 (PMC9552458; doi:10.1186/s12884-022-05080-5)
Supplement: Supplementary file 2 — Additional file 2. Maternal Health Service Coverage Index (MHSI) values at sub-national level in India based four different scenarios for base year (2017-2018) and reference year (2019-2020). [file 12884_2022_5080_MOESM2_ESM.pdf]

Additional file 2

Additional file 2.pdf

Title: Maternal Health Service Coverage Index (MHSI) values at sub-national level in India based four different scenarios for base year (2017-2018) and reference year (2019-2020)

State/UTs	Base year: 2017-2018				Reference year: 2019-2020			
	**Scenario I**	**Scenario II**	**Scenario III**	**Scenario IV**	**Scenario I**	**Scenario II**	**Scenario III**	**Scenario IV**
**North zone**								
Chandigarh	0.370	0.244	0.244	0.183	0.470	0.377	0.377	0.331
Delhi	0.437	0.418	0.412	0.332	0.439	0.436	0.432	0.312
Haryana	0.549	0.522	0.510	0.404	0.530	0.505	0.500	0.398
Himachal Pradesh	0.608	0.557	0.550	0.483	0.699	0.646	0.645	0.575
Jammu& Kashmir	0.505	0.478	0.451	0.342	0.499	0.486	0.468	0.376
Punjab	0.645	0.621	0.610	0.504	0.663	0.642	0.635	0.545
Rajasthan	0.309	0.302	0.291	0.234	0.345	0.271	0.265	0.224
Uttar Pradesh	0.318	0.315	0.291	0.211	0.436	0.422	0.405	0.316
Uttarakhand	0.419	0.411	0.396	0.307	0.433	0.425	0.408	0.311
**West zone**								
Chhattisgarh	0.597	0.589	0.560	0.504	0.572	0.559	0.536	0.469
Dadra & Nagar Haveli	0.556	0.520	0.518	0.412	0.663	0.610	0.606	0.477
Goa	0.564	0.544	0.542	0.430	0.660	0.641	0.640	0.555
Gujarat	0.568	0.547	0.516	0.402	0.654	0.641	0.618	0.490
Madhya Pradesh	0.356	0.337	0.318	0.241	0.337	0.274	0.262	0.207
Maharashtra	0.540	0.529	0.509	0.422	0.588	0.572	0.561	0.477
**East & north east zone**								
Andaman & Nicobar Island	0.644	0.624	0.620	0.528	0.669	0.628	0.621	0.532
Arunachal Pradesh	0.378	0.370	0.369	0.289	0.337	0.321	0.320	0.252
Assam	0.533	0.508	0.462	0.384	0.626	0.594	0.561	0.478
Bihar	0.334	0.328	0.297	0.201	0.369	0.361	0.327	0.231
Jharkhand	0.410	0.407	0.374	0.277	0.467	0.462	0.443	0.332
Manipur	0.438	0.434	0.423	0.310	0.426	0.426	0.420	0.305
Meghalaya	0.340	0.318	0.314	0.258	0.340	0.327	0.325	0.266
Mizoram	0.481	0.454	0.446	0.321	0.346	0.305	0.304	0.248
Nagaland	0.371	0.360	0.358	0.234	0.372	0.370	0.368	0.247
Odisha	0.591	0.557	0.533	0.456	0.692	0.659	0.647	0.570
Sikkim	0.629	0.602	0.579	0.488	0.628	0.614	0.605	0.492
Tripura	0.479	0.451	0.446	0.263	0.504	0.482	0.478	0.269
West Bengal	0.584	0.546	0.502	0.383	0.614	0.583	0.565	0.394
**South zone**								
Andhra Pradesh	0.605	0.594	0.574	0.454	0.660	0.651	0.638	0.516
Karnataka	0.712	0.678	0.672	0.567	0.723	0.692	0.690	0.596
Kerala	0.858	0.711	0.688	0.545	0.748	0.689	0.681	0.529
Puducherry	0.546	0.532	0.523	0.466	0.388	0.371	0.368	0.331
Tamil Nadu	0.462	0.194	0.192	0.180	0.476	0.202	0.201	0.189
Telangana	0.540	0.530	0.499	0.392	0.472	0.452	0.436	0.341
