# Supplementary material for: Measurement and mapping of maternal health service coverage through a novel composite index: a sub-national level analysis in India
Source: BMC Pregnancy Childbirth. 2022 Oct 10;22:761. doi: 10.1186/s12884-022-05080-5 (PMC9552458; doi:10.1186/s12884-022-05080-5)
Supplement: Supplementary file 3 — Additional file 3. Incremental change of MHSI values and categorization of states in base year and reference year (Scenario II). [file 12884_2022_5080_MOESM3_ESM.pdf]

Additional file 3

Additional file 3.pdf

Title: Incremental change of MHSI values and categorization of states in base year and reference year (***Scenario II***)

**State/UTs**	**MHSI Values in Base Year (2017-18)**		**MHSI Values in Reference year (2019-20)**		**Incremental Changes in MHSI**	
	**Index values**	**Category**	**Index value**	**Category**	**Change in value**	**Change in category**
			**North zone**			
Chandigarh*	0.244	Low	0.377	Low	0.133	→
Delhi*	0.418	Medium	0.436	Medium	0.019	→
Haryana	0.522	Medium	0.505	Medium	-0.017	←
Himachal Pradesh	0.557	High	0.646	High	0.089	→
Jammu& Kashmir	0.478	Medium	0.486	Medium	0.008	→
Punjab	0.621	High	0.642	High	0.021	→
Rajasthan	0.302	Low	0.271	Low	-0.031	←
Uttar Pradesh	0.315	Low	0.422	Medium	0.107	↑
Uttarakhand	0.411	Low	0.425	Medium	0.015	↑
			**West zone**			
Chhattisgarh	0.589	High	0.559	High	-0.030	←
Dadra & Nagar Haveli*	0.520	Medium	0.610	High	0.090	↑
Goa	0.544	Medium	0.641	High	0.098	↑
Gujarat	0.547	High	0.641	High	0.094	→
Madhya Pradesh	0.337	Low	0.274	Low	-0.062	←
Maharashtra	0.529	Medium	0.572	High	0.043	↑
			**East & north east zone**			
Andaman & Nicobar Island*	0.624	High	0.628	High	0.005	→
Arunachal Pradesh	0.370	Low	0.321	Low	-0.049	←
Assam	0.508	Medium	0.594	High	0.085	↑
Bihar	0.328	Low	0.361	Low	0.033	→
Jharkhand	0.407	Low	0.462	Medium	0.054	↑
Manipur	0.434	Medium	0.426	Medium	-0.008	←
Meghalaya	0.318	Low	0.327	Low	0.009	→
Mizoram	0.454	Medium	0.305	Low	-0.149	↓
Nagaland	0.360	Low	0.370	Low	0.010	→
Odisha	0.557	High	0.659	High	0.102	→
Sikkim	0.602	High	0.614	High	0.011	→
Tripura	0.451	Medium	0.482	Medium	0.031	→
West Bengal	0.546	High	0.583	High	0.038	→
			**South zone**			
Andhra Pradesh	0.594	High	0.651	High	0.057	→
Karnataka	0.678	High	0.692	High	0.014	→
Kerala	0.711	High	0.689	High	-0.021	←
Puducherry*	0.532	Medium	0.371	Low	-0.161	↓
Tamil Nadu	0.194	Low	0.202	Low	0.008	→

Telangana	0.530	Medium	0.452	Medium	-0.078	←

↑ Upward transition from a lower category to higher; → No change in category but MHSI value increased; → No change in category but MHSI value decreased; ↓ Downward transition from a higher category to lower.

**Denotes Union Territory (UT).*
