# Supplementary material for: Measurement and mapping of maternal health service coverage through a novel composite index: a sub-national level analysis in India
Source: BMC Pregnancy Childbirth. 2022 Oct 10;22:761. doi: 10.1186/s12884-022-05080-5 (PMC9552458; doi:10.1186/s12884-022-05080-5)
Supplement: Supplementary file 4 — Additional file 4. Incremental change of MHSI values and categorization of states in base year and reference year (Scenario III). [file 12884_2022_5080_MOESM4_ESM.pdf]

Additional file 4

Additional file 4.pdf

Title: Incremental change of MHSI values and categorization of states in base year and reference year (***Scenario III***)

State/UTs	MHSI Values in Base Year (2017-18)		MHSI Values in Reference year (2019-20)		Incremental Changes in MHSI	
	Index value	Category	Index value	Category	Change in value	Change in category
**North zone**						
Chandigarh*	0.451	Medium	0.377	Low	-0.075	↓
Delhi*	0.374	Low	0.432	Medium	0.059	↑
Haryana	0.672	High	0.500	Medium	-0.172	↓
Himachal Pradesh	0.688	High	0.645	High	-0.042	←
Jammu& Kashmir	0.318	Low	0.468	Medium	0.151	↑
Punjab	0.509	Medium	0.635	High	0.127	↑
Rajasthan	0.423	Medium	0.265	Low	-0.158	↓
Uttar Pradesh	0.314	Low	0.405	Low	0.091	→
Uttarakhand	0.446	Medium	0.408	Medium	-0.037	←
**West zone**						
Chhattisgarh	0.192	Low	0.536	High	0.344	↑
Dadra & Nagar Haveli*	0.499	Medium	0.606	High	0.107	↑
Goa	0.446	Medium	0.640	High	0.194	↑
Gujarat	0.291	Low	0.618	High	0.328	↑
Madhya Pradesh	0.396	Low	0.262	Low	-0.134	←
Maharashtra	0.502	Medium	0.561	High	0.059	↑
**East & north east zone**						
Andaman & Nicobar Island*	0.620	High	0.621	High	0.001	→
Arunachal Pradesh	0.574	High	0.320	Low	-0.254	↓
Assam	0.369	Low	0.561	High	0.192	↑
Bihar	0.462	Medium	0.327	Low	-0.135	↓
Jharkhand	0.297	Low	0.443	Medium	0.146	↑
Manipur	0.244	Low	0.420	Medium	0.176	↑
Meghalaya	0.560	High	0.325	Low	-0.236	↓
Mizoram	0.518	Medium	0.304	Low	-0.215	↓
Nagaland	0.412	Medium	0.368	Low	-0.045	↓
Odisha	0.542	High	0.647	High	0.104	→
Sikkim	0.516	Medium	0.605	High	0.089	↑
Tripura	0.510	Medium	0.478	Medium	-0.031	←
West Bengal	0.550	High	0.565	High	0.014	→
**South zone**						
Andhra Pradesh	0.358	Low	0.638	High	0.280	↑
Karnataka	0.533	High	0.690	High	0.157	→
Kerala	0.523	High	0.681	High	0.157	→
Puducherry*	0.610	High	0.368	Low	-0.243	↓
Tamil Nadu	0.291	Low	0.201	Low	-0.090	←

Telangana	0.579	High	0.436	Medium	-0.142	↓

↑ *Upward transition from a lower category to higher;* → *No change in category but MHSI value increased;* → *No change in category but MHSI value decreased;* ↓ *Downward transition from a higher category to lower.*

**Denotes Union Territory (UT).*
