# Supplementary material for: Measurement and mapping of maternal health service coverage through a novel composite index: a sub-national level analysis in India
Source: BMC Pregnancy Childbirth. 2022 Oct 10;22:761. doi: 10.1186/s12884-022-05080-5 (PMC9552458; doi:10.1186/s12884-022-05080-5)
Supplement: Supplementary file 5 — Additional file 5. Formula used in the construction of MHSI in four different scenarios. [file 12884_2022_5080_MOESM5_ESM.pdf]

Additional file 5

Additional file 5.pdf

Title: Formula used in the construction of MHSI in four different scenarios

	Step 1	Step 2	Step 3
Scenario I	Arithmetic mean approach<br>$\frac{(ANC1\_1 + ANC1\_2)}{2} = ANC1$<br>$\frac{(ANC2\_1+ANC2\_2+ ANC2\_3+ANC2\_4+ANC2\_5)}{5} = ANC2$<br>$\frac{(ANC3\_1+ANC3\_2+ANC3\_3)}{3} = ANC3$<br>$\frac{(INC\_1+INC\_2+ INC\_3+INC\_4)}{4} = INC$<br>$PNC_1 = PNC$	Arithmetic mean approach<br>$\frac{(ANC1 + ANC2+ ANC3)}{3} = ANC$	Arithmetic mean approach<br>$\frac{(ANC + INC+ PNC)}{3} = MHSI$
Scenario II	Arithmetic mean approach<br>$\frac{(ANC1\_1 + ANC1\_2)}{2} = ANC1$<br>$\frac{(ANC2\_1+ANC2\_2+ ANC2\_3+ANC2\_4+ANC2\_5)}{5} = ANC2$<br>$\frac{(ANC3\_1+ANC3\_2+ANC3\_3)}{3} = ANC3$<br>$\frac{(INC\_1+INC\_2+ INC\_3+INC\_4)}{4} = INC$<br>$PNC_1 = PNC$	Arithmetic mean approach<br>$\frac{(ANC1 + ANC2+ ANC3)}{3} = ANC$	Geometric mean approach<br>$\sqrt[3]{ANC \times INC \times PNC} = MHSI$
Scenario III	Arithmetic mean approach<br>$\frac{(ANC1\_1 + ANC1\_2)}{2} = ANC1$<br>$\frac{(ANC2\_1+ANC2\_2+ ANC2\_3+ANC2\_4+ANC2\_5)}{5} = ANC2$<br>$\frac{(ANC3\_1+ANC3\_2+ANC3\_3)}{3} = ANC3$<br>$\frac{(INC\_1+INC\_2+ INC\_3+INC\_4)}{4} = INC$<br>$PNC_1 = PNC$	Geometric mean approach<br>$\sqrt[3]{ANC1 \times ANC2 \times ANC3} = ANC$	Geometric mean approach<br>$\sqrt[3]{ANC \times INC \times PNC} = MHSI$
Scenario IV	Geometric mean approach<br>$\sqrt[2]{ANC1\_1 \times ANC1\_2} = ANC1$<br>$\sqrt[5]{ANC2\_1 \times ANC2\_2 \times ANC2\_3 \times ANC2\_4 \times ANC2\_5} = ANC2$<br>$\sqrt[3]{ANC3\_1 \times ANC3\_2 \times ANC3\_3} = ANC3$<br>$\sqrt[4]{INC\_1 \times INC\_2 \times INC\_3 \times INC\_4} = INC$<br>$PNC_1 = PNC$	Geometric mean approach<br>$\sqrt[3]{ANC1 \times ANC2 \times ANC3} = ANC$	Geometric mean approach<br>$\sqrt[3]{ANC \times INC \times PNC} = MHSI$
